# Differential Responses of Human Dendritic Cells to Live or Inactivated *Staphylococcus aureus*: Impact on Cytokine Production and T Helper Expansion

**DOI:** 10.3389/fimmu.2019.02622

**Published:** 2019-11-12

**Authors:** Melania Cruciani, Silvia Sandini, Marilena P. Etna, Elena Giacomini, Romina Camilli, Martina Severa, Fabiana Rizzo, Fabio Bagnoli, John Hiscott, Eliana M. Coccia

**Affiliations:** ^1^Department of Infectious Diseases, Istituto Superiore di Sanità, Rome, Italy; ^2^GlaxoSmithKline (GSK) Srl, Siena, Italy; ^3^Pasteur Laboratory, Istituto Pasteur-Fondazione Cenci Bolognetti, Rome, Italy

**Keywords:** DC, *S. aureus*, cytokines, signaling pathway, T cell response

## Abstract

Understanding *Staphylococcus aureus* (*S. aureus*)–host immune system interaction is crucial to meet the tremendous medical need associated with this life-threatening bacterial infection. Given the crucial role of dendritic cells (DC) in dictating immune responses upon microbial challenge, we investigated how the bacterial viability and the conservation of structural integrity influence the response of human DC to *S. aureus*. To this end, human primary DC were stimulated with the methicillin-resistant *S. aureus* USA300 live strain, USA300 inactivated by heat (HI), ultraviolet irradiation (UVI), or paraformaldehyde treatment (PFAI) and subsequently analyzed for cell phenotype and immune-modulatory properties. Although no differences in terms of DC viability and maturation were observed when DC were stimulated with live or inactivated bacteria, the production of IL-12, IL-23, and other cytokines differed significantly. The Th1 and Th17 expansion was also more pronounced in response to live vs. inactivated *S. aureus*. Interestingly, cytokine production in DC treated with live and inactivated USA300 required phagocytosis, whereas blocking endosomal Toll-like receptor signaling mainly reduced the cytokine release by live and HI USA300. A further analysis of IFN-β signaling revealed the induction of a cyclic GMP-AMP synthase stimulator of interferon genes (cGAS-STING)-independent and IRF3-dependent signaling pathway(s) in UVI-stimulated DC. This study underscores the capacity of human DC to discriminate between live and inactivated *S. aureus* and, further, indicates that DC may represent a valuable experimental setting to test different inactivation methods with regard to the retention of *S. aureus* immunoregulatory properties. These and further insights may be useful for the development of novel therapeutic and prophylactic anti-*S. aureus* vaccine strategies.

## Introduction

*Staphylococcus aureus* (*S. aureus*) is an opportunistic pathogen that causes a wide spectrum of infections, ranging from superficial skin lesions to life-threatening conditions, such as sepsis, endocarditis, and pneumonia (1, 2). The large numbers of nosocomial and community-acquired infections make *S. aureus* a serious public health problem and, consequently, a major burden on society worldwide (3). In addition, the resistance or limited effectiveness of antibiotic treatment, as well as the lack of an effective vaccine against these bacteria reveals a global unmet medical need for effective therapies.

Analysis of immune response against *S. aureus* has revealed a complex and multifaceted process (4, 5). The wide repertoire of *S. aureus* virulence factors triggers multiple pattern recognition receptor (PRR) pathways, leading to the activation of different innate and, subsequently, adaptive immune responses. Activation of Toll-like receptors (TLRs) such as TLR2 (6), TLR9 (7), and TLR8 (8) after *S. aureus* infection has been demonstrated in human and mouse settings. In addition, *S. aureus* can escape from host cell endosomes to the cytoplasm (9), where it can activate cytosolic sensors, including the nucleotide oligomerization domain 2 (NOD-2) (10), the NOD-like receptor P3 (NLRP3) (11), Absent in melanoma 2 (AIM2) (12), and stimulator of interferon (IFN) genes (STING) (13). In different cell types, infection with *S. aureus* induces type I IFN signaling through the activation of diverse PRRs (7, 14). Recently, Scumpia et al. showed that both TLR and STING pathways are activated a few hours after macrophage infection with live *S. aureus* and compete for the regulation of ~95% of the induced genes; in particular, TLR signaling predominantly activated a proinflammatory program while STING signaling activated an antiviral/type I IFN response (13). Conversely, heat-killed bacterium activated mainly TLR signaling (13). This differential response may be related to the production of signaling nucleotides—cyclic di-AMP (c-di-AMP) synthesized in response to infection with live bacteria—that are able to activate the pro-inflammatory cGAS–STING–IRF3 response leading to type I IFN production (15, 16). However, the relative contribution of each pathway in orchestrating the immune response against *S. aureus* is not fully understood and is complicated by the existence of many bacterial strains and infection routes.

Dendritic cells (DC) are key components of the immune system for their extraordinary capacity to initiate primary immune responses and stimulate naïve T cells (17). DC play an important role in orchestrating and regulating immune responses against pathogens, including *S. aureus* (18, 19). In this respect, we recently demonstrated that the virulence factors Esx secreted by live *S. aureus* modulate human DC functions and their capacity to support a Th1/Th17 response (20). These results illustrated how human DC may sense small differences in *S. aureus* virulence to dynamically instruct both innate and adaptive immunity.

In the present study, we further interrogated the DC experimental setting to understand how the viability and the structural integrity of *S. aureus* may influence the interaction with human DC and, in turn, the outcome of the Th response. Indeed, because of the *vita*-pathogen associated molecular patterns (PAMPs)—structures associated only with live microorganisms (21)—innate immune responses can discriminate between live and dead microbes and rapidly evaluate the severity of the infectious threat and modulate the magnitude and the quality of the immune responses (22–24).

An *in vitro* model, based on human monocyte-derived DC, was used to analyze differences in the response to live or inactivated *S. aureus* preparations in terms of DC phenotype, immune-modulatory properties, and regulation of cytokine production. This study highlights important aspects of the complex and multifaceted interplay of different innate immune signaling pathways in human DC in response to the interaction with live and differently inactivated *S. aureus*, which could be exploited to design novel therapeutic and prophylactic anti-*S. aureus* vaccine strategies.

## Materials and Methods

### Antibodies and Other Reagents

Monoclonal antibodies (Abs), specific for cluster of differentiation (CD)1a, CD14, CD38, CD86, CD83, HLA-DR, CD40, IgG1, and IgG2a (BD Bioscience, San Diego, CA, USA), were directly conjugated to fluorescein isothiocyanate (FITC) or phycoerythrin (PE). To exclude dead cells from the analysis, Fixable Viability Dye eFluor®780 (FvDye) (eBioscience, San Diego, CA, USA) was used. For immunoblotting analysis, rabbit anti-STING (Cell Signaling, Danvers, MA, USA # 2775), anti-IRF3 (Santa Cruz, Santa Cruz, TX, USA # sc-9082), anti-IRF7 (Santa Cruz, # sc-9083), anti-STAT1 (BD Bioscience, # 610186), anti-phospho STAT1 (Cell Signaling Technology, Leiden, The Netherlands, # 7649), anti-STAT2 (BD Transduction Laboratories, # 610188), anti-phospho STAT2 (R&D Systems, Minneapolis, MN, USA, MAB2890), mouse anti-actin (Sigma-Aldrich, St. Louis, MO, USA #A0483), and horseradish peroxidase-conjugated secondary antibody anti mouse (Santa Cruz, # sc-2005) and anti rabbit (Santa Cruz, # sc-2004) were used. For phagocytosis and phagosomal acidification experiments, cytochalasin D 5 μM (Sigma-Aldrich, # C8723) and chloroquine 2 μM (Sigma-Aldrich, # C6628) were used.

### Bacterial USA300 Growth and Inactivation Conditions

Briefly, *S. aureus* USA300 was grown in tryptic soy broth (TSB, BD Bioscience # BA-25107.05) overnight at 37°C. The next day, bacterial broth was diluted 1:100 in fresh TSB, cultured until the exponential phase of growth (OD_600_ of 0.6), and then washed in RPMI 1640 and resuspended in RPMI 1640 supplemented with l-glutamine (2 mM) and 15% fetal bovine serum (FBS) for DC infection. To inactivate USA300, 10 ml of bacterial culture (0.6 OD_600_) was washed in PBS and either treated with UV-irradiation (254 nm) for 40 min on ice (UV-inactivated, UVI), heat-inactivated at 100°C for 15 min (HI) or fixed with 4% paraformaldehyde (PFA-inactivated, PFAI) (Panreac Quimica, Castellar del Valles, ES) for 30 min at room temperature (RT), and then washed three times with PBS. To confirm the lack of viable bacteria following UV irradiation, heat killing, or PFA fixation, 200 μl of the undiluted bacterial suspension was plated on tryptic soy agar (TSA, Oxoid, Basingstoke, Hants, UK) and incubated at 37°C overnight. The absence of growth indicated the efficacy of the inactivation procedures.

### DC Preparation and Stimulation

Istituto Superiore di Sanità (ISS) Review Board approved the present research project (CE/13/387). Informed consent was obtained from all donors before collecting the blood samples. No specific analysis on *S. aureus* infectious status was performed on blood samples given for research studies since the agreement signed by ISS and the Blood Donation Center does not allow the monitoring of specific infectious diseases in addition to those routinely performed within the serology testing for suitability of blood donation. DC were prepared as previously described (25). DC were generated by culturing monocytes with 50 ng/ml GM-CSF (R&D Systems, Minneapolis, MN, USA) and 200 U/ml of IL-4 (Miltenyi, Bergisch Gladbach, DE) for 5 days at 0.5 × 10^6^ cells/ml in RPMI 1640 (BioWhittaker Europe, Verviers, BE) supplemented with l-glutamine (2 mM) (Lonza, Basel, CH) and 15% FBS (Lonza). At day 5, cells were tested for their differentiation status by evaluating CD1a expression (>90% CD1a^+^- as apex; “CD14^−^”) and lack of CD14 (>95% CD14). Before stimulation, the medium was replaced with RPMI supplemented only with l-glutamine (2 mM) and 15% FBS. Cytokine deprivation did not affect DC survival rate, which was >90%. For live *S. aureus* infection, DC were infected as previously described (20), using a multiplicity of infection (MOI) of 0.1 bacterium/cell. For treatment with inactivated *S. aureus* preparations, a dose response with inactivated bacteria was used, monitoring CD86 expression on the surface of stimulated DC (data not shown). An MOI of 1 inactivated bacterium/cell was the chosen dose leading to a comparable stimulation to that observed in DC infected with live bacteria and was then used in all experiments. To assess the role of phagocytosis in the induction of cytokine response, prior to the stimulation with live and inactivated USA300 strain, DC were pre-treated with 5 μM Cytochalasin D for 30 min. Inhibition of endosomal acidification was performed by pre-treating DC with 2 μM chloroquine for 30 min before bacterial stimulation.

### Flow Cytometry Analysis

Cells (10^5^) were washed once in phosphate buffered saline (PBS) (Lonza) containing 2% FBS and incubated with indicated monoclonal Abs at 4°C for 30 min. DC were then washed and fixed with 2% formaldehyde (Panreac Quimica) before analysis on a Gallios cytometer (Beckman Coulter, Brea, CA, USA). A total of 30,000 events were analyzed per sample in FvDye negative live cells. In viable DC, the expression of cell surface molecules was evaluated using the median fluorescence intensity (MFI) after subtraction of the values of the isotype Ab controls.

### Cell Viability Detection

Cell viability was analyzed by using FvDye according to manufacturing protocols. Twenty-four hours post infection, DC were stained with FvDye as previously described (26). Finally, cells were fixed overnight with 4% formaldehyde before analysis on a Gallios cytometer (Beckman Coulter). Data were analyzed by Kaluza software (Beckman Coulter).

### T Cell Response

Total CD4^+^ T cells were isolated from autologous frozen peripheral blood mononuclear cells by indirect magnetic sorting with a CD4^+^ T-cell isolation kit (Miltenyi), as previously described (26). Purified cells were plated in 96-well U-bottomed tissue culture plates at the density of 0.4 × 10^6^ cells/ml with DC previously stimulated for 24 h with the live and inactivated *S. aureus* at a density of 0.4 × 10^5^/ml (ratio 1 DC: 10 CD4^+^ T cells). At day 5, supernatants were harvested for IFN-γ and IL-17 detection.

### Cytokine Determination

Supernatants of DC cultures were harvested 24 h after stimulation with live and inactivated *S. aureus*, filtered (0.2 μm), and stored at −80°C. The production of IL-12, TNF-α, IL-10, IL-1β, IL-6, and IL-8 was measured by human Inflammatory Cytokine kit (Cytometric Bead Array, CBA, BD Bioscience). Release of IL-23, IFN-γ, and IL-17 was instead assayed by specific ELISA kits (R&D Systems) or by human Th1/Th2 Cytokine kit (Cytometric Bead Array, CBA, BD Bioscience).

### RNA Isolation and Quantitative Real-Time PCR

Total RNA was extracted from DC (1 × 10^6^) using TRIzol Reagent (Thermo Fisher Scientific, Palm beach, FL, USA) following the manufacturer's recommendations. Reverse transcriptions were performed as previously described (25). Quantitative PCR assays were performed at least in duplicate using the Platinum Taq DNA Polymerase (Invitrogen Life Technologies Frederick, MD) and the SYBR Green I (Lonza) on a LightCycler (Roche Diagnostics, Basel, CH). Primer pairs used to analyze GAPDH, IFN-β, and IFN-αs expression have been previously described (27). Transcript expression was normalized to the GAPDH level using the Equation 2^−Δ*Ct*^.

### Immunoblot Analysis

Western blotting was performed as previously described (25). Briefly, 25 μg of total protein extracts was separated on 10% SDS-PAGE gel and blotted onto nitrocellulose membranes (Merck–Millipore, Darmstadt, DE). Blots were incubated with rabbit polyclonal Abs against STING, IRF3, IRF7, STAT-1, and STAT-2. Detection was achieved using anti-rabbit horseradish peroxidase-conjugated secondary Ab (Santa Cruz), and visualized with Enhanced Chemiluminescence plus kit (GE Healthcare Bio-Sciences, Pittsburgh, PA, USA). A ChemiDoc XRS (Bio-Rad, Hercules, CA, USA) instrument and ImageLab software (Bio-Rad) were used to reveal and analyze the chemiluminescence signal. β-Actin levels were analyzed by mouse anti-β-actin Ab to verify the loaded protein amount.

### Statistical Analysis

Statistical analysis was carried out using a two-tailed Student's *t*-test for paired data. A *p*-value < 0.05 was considered statistically significant.

## Results

### Live or Inactivated *S. aureus*-Stimulated DC Drive a Differential Expansion of Th1 and Th17 Cells

**Figure 1:**
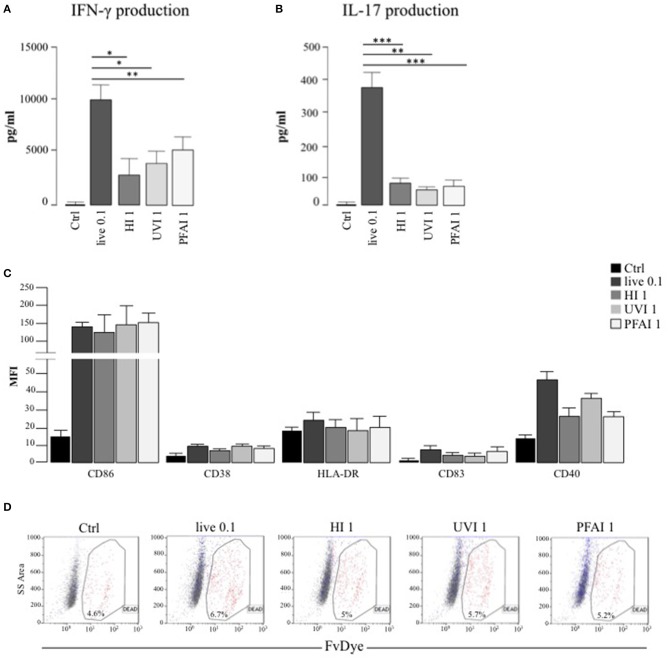
DC response to live and inactivated *S. aureus*. **(A,B)** Expansion of IFN-γ and IL-17 producing T cells driven by live and inactivated *S. aureus*-stimulated DC. Untreated DC (Ctrl) and DC stimulated for 24 h with live and differentially inactivated USA300 strain were co-cultured with autologous total CD4^+^ T cells for 5 days. **(A)** The level of IFN-γ was measured by human Th1/Th2 Cytokine array kit in harvested supernatants. **(B)** IL-17 secretion was instead evaluated by ELISA. The results represent means ± SEM of four independent experiments (*p ≤ 0.05; **p ≤ 0.01; ***p ≤ 0.001). **(C,D)** Analysis of DC maturation and viability in response to live, HI-, UVI-, or PFAI-USA300 stimulation. DC were left untreated (Ctrl) or stimulated for 24 h with live and differentially inactivated USA300 strain. **(C)** Surface expression of the indicated molecules was evaluated by cytofluorimetric analysis in three independent experiments and graphed by calculating the mean fluorescence intensity (MFI) after the subtraction of the isotype Ab controls. Mean MFI ± SEM are shown. **(D)** Cell viability was evaluated by DC staining with FvDye. Numbers in the dot plots correspond to the percentage of dead cells. A representative experiment, out of three independent experiments performed that yielded similar results, is shown.

We previously demonstrated that DC respond to *S. aureus* infection by acquiring a mature phenotype required for the conditioning of Th1/Th17 response (20). Here, we extend our analysis to the characterization of how different *S. aureus* inactivation methods impact the DC immune phenotype and regulatory properties. After the identification of the optimal *S. aureus* MOI and stimulation doses with inactivated bacteria (see Materials and Methods), we assayed the ability of DC stimulated with either live or killed bacteria to expand IFN-γ and IL-17 producing CD4^+^ T lymphocytes in a mixed lymphocyte reaction (MLR) setting. Interestingly, we observed a 2-fold and 4-fold higher production of IFN-γ and IL-17 in MLR cultures of DC infected with live *S. aureus* USA300, compared to non-replicating bacteria, respectively (Figures 1A,B). A Th1 promoting phenotype was conferred by killed *S. aureus* USA300, in particular by UVI and PFAI preparations (Figure 1A), whereas all inactivated bacteria poorly induced IL-17 production (Figure 1B). This differential response was not related to the maturation status of DC cultures, since the cytofluorimetric analysis of CD86, CD83, HLA-DR, CD40, and CD38 expression demonstrated that all markers were induced to a similar extent on DC surface after 24 h stimulation with live, HI-, UVI-, or PFAI-USA300 (Figure 1C). In addition no effect of different bacterial preparations on cell viability was observed (Figure 1D).

### Induction of a Different Cytokine Profile in DC Stimulated With Live or Inactivated *S. aureus*

**Figure 2:**
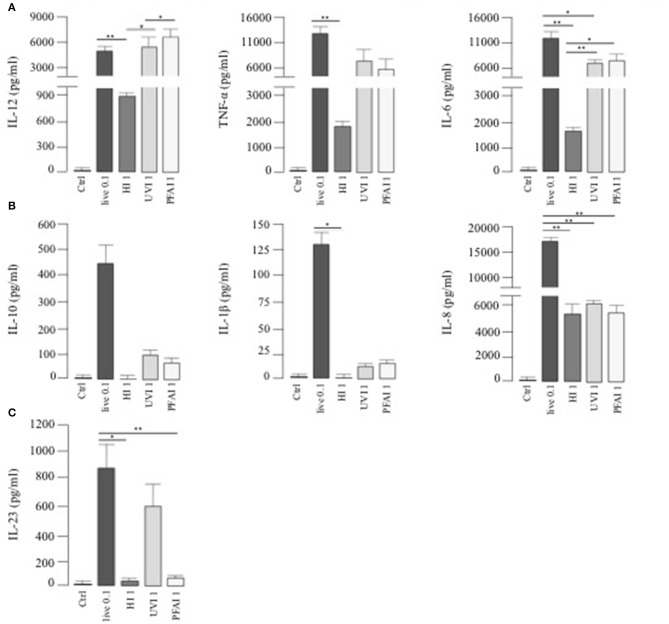
Cytokine release by DC stimulated with live, HI-, UVI-, and PFAI-USA300. DC were left untreated (Ctrl) or stimulated for 24 h with live and differentially inactivated USA300 strain. The secretion of IL-12, TNF-α, IL-6 **(A)**, IL-10, IL-1β, IL-8 **(B)**, and IL-23 **(C)** was measured in DC culture supernatants. The results represent means ± SEM of four independent experiments (*p ≤ 0.05; **p ≤ 0.01).

To investigate whether a different profile of cytokine production could be responsible for the Th1/Th17 response, we analyzed cytokine release from DC stimulated for 24 h with live or HI, UVI-, and PFAI-USA300. A robust production of IL-12, TNF-α, and IL-6 was observed in response to live and UVI and PFAI *S. aureus* preparations. DC stimulated by HI USA300 produced 6- to 10-fold lower of those cytokines (Figure 2A). IL-10, IL-1β, and the chemokine IL-8 were mainly released from DC infected with live USA300 (Figure 2B). We also observed that IL-23 induction occurred with live and UVI bacteria, while no production was detected in DC cultured in the presence of HI- and PFAI *S. aureus* (Figure 2C).

### Characterization of Type I IFN Expression and Intracellular Pathway in DC Treated With Live or Inactivated *S. aureus*

**Figure 3:**
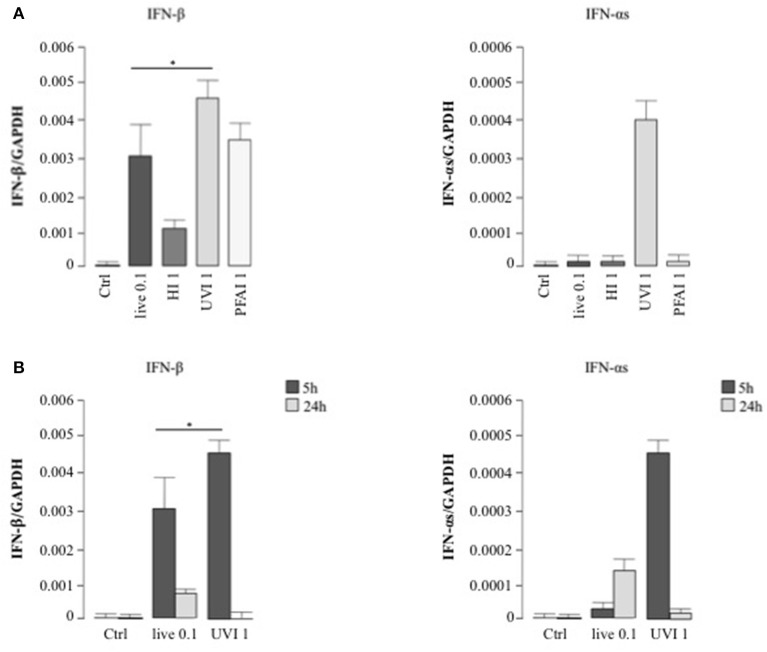
Type I IFN expression in DC stimulated with live and inactivated USA300 preparations. DC were left untreated (Ctrl) or stimulated for 5 h with live, HI, UVI, and PFAI *S. aureus* **(A)** and for 24 h with live and UVI *S. aureus* **(B)**. The expression of IFN-β and IFN-αs was evaluated by real-time quantitative RT-PCR. mRNA levels were normalized by the 2^−Δ*Ct*^ formula using GAPDH as housekeeping gene. The results represent means ± SEM of four independent experiments (*p ≤ 0.05).

We extended this analysis to type I IFNs (mainly IFN-αs and IFN-β), pivotal cytokines in the regulation of innate immune response against pathogens (28). Interestingly, stimulation with UVI *S. aureus* induced a significantly higher IFN-β mRNA level than observed in cells infected with live bacteria (Figure 3A). HI *S. aureus* induced 3-fold less IFN-β expression with respect to PFAI and live treatments (Figure 3A). With regard to IFN-α, only treatment with UVI *S. aureus* induced IFN-α expression after 5 h (Figure 3A), although at 24 h, live bacteria also drove IFN-α expression (Figure 3B).

**Figure 4:**
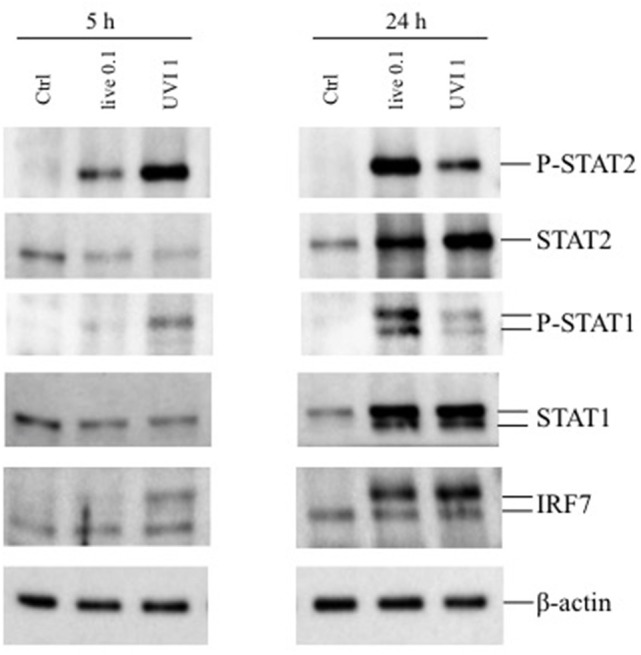
Expression of phosphorylated STAT1/2 and IRF7 in DC stimulated with live and UVI-USA300. DC were left untreated (Ctrl) or stimulated with live and UVI-USA300 for 5 and 24 h. Cell lysates were analyzed by Western blot to detect the expression and the phosphorylation of the indicated proteins. β-actin levels were analyzed as control for protein loading.

Next, the activation of STAT transcription factors involved in type I IFN signaling—STAT1 and STAT2—as well as expression of the IFN-inducible gene IRF7 were analyzed in DC stimulated for 5 and 24 h with live or UVI *S. aureus* to prove the functionality of the type I IFN expression analyzed by real-time PCR in Figure 3B. After 5 h stimulation, STAT1 and STAT2 displayed an increased level of phosphorylation in UVI-treated DC, compared to those present in DC cultures infected with live *S. aureus*. Similarly, IRF7 expression was stimulated by live *S. aureus* (Figure 4). Conversely, at 24 h, an inverse pattern of expression was observed, with higher activation in live USA300-challenged cells, which correlates with the long-lasting type I IFN induction in these cultures (as seen in Figure 3B).

### DC Response Is Triggered by Live and Inactivated *S. aureus* Through the Activation of Different Intracellular Signaling Pathways

**Figure 5:**
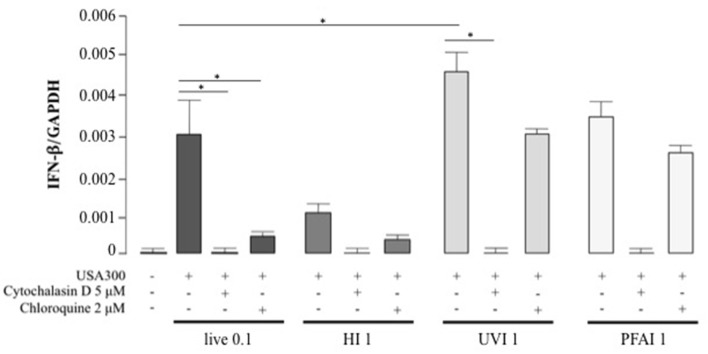
Effect of phagocytosis and phagosomal acidification inhibition on IFN-β expression in DC stimulated with live, HI, UVI, and PFAI USA300. DC were pre-treated with 5 μM Cytochalasin D or with 2 μM Chloroquine for 30 min and then stimulated for 5 h with live and inactivated USA300 strain. The expression of IFN-β was evaluated by real-time quantitative RT-PCR. mRNA levels were normalized by the 2^−Δ*Ct*^ formula using GAPDH as housekeeping gene. The results represent means ± SEM of four independent experiments (*p ≤ 0.05).

Cytokine expression in response to pathogens is triggered by different signaling pathways, activated by either membrane or intracellular receptors as well as cytosolic sensors (29, 30). To determine if the cytokine profiles were driven by extracellular sensing or by phagocytosis, DC were pre-treated with cytochalasin D, an inhibitor of actin polymerization that blocks bacterial internalization, prior to stimulation with live and inactivated *S. aureus* (Figure 5 and Table 1). IFN-β expression was completely abrogated in DC stimulated with live or different inactivated USA300 preparations when cytochalasin D was added to DC cultures (Figure 5), indicating that internalization and DC interaction with surface molecules were necessary to trigger IFN-β expression.

**Table 1:** Effect of phagocytosis and phagosomal acidification inhibition on DC cytokine expression induced by live and inactivated USA300 preparations.

	**Treatment**	**pg/ml**	**+Cytochalasin D (5 μM)**	**+ Chloroquine (2 μM)**
IL-12	USA300 live 0.1	4,668 ± 335	6.3 ± 61^**^	838 ± 5901^**^
	USA300 HI 1	1,038 ± 30	0.3 ± 0.31^***^	239 ± 51^***^
	USA300 UV 1	5,342 ± 931	Not det	3,960 ± 2,294
	USA300 PFA 1	6,704 ±2,768	3 ± 3	2,414 ± 2,052
TNF-α	USA300 live 0.1	13,013 ± 1,881	83 ± 451^**^	2,138 ± 7211^**^
	USA300 HI 1	872 ± 507	16 ± 3	411 ± 208
	USA300 UV 1	6,473 ± 2,432	21 ± 7	3,798 ± 55
	USA300 PFA 1	6,074 ± 1,196	27 ± 141^*^	2,279 ± 8451^**^
IL-6	USA300 live 0.1	11,465 ± 2,518	55 ± 111^**^	2,088 ± 1,217
	USA300 HI 1	1,793 ± 245	26 ± 91^**^	572 ± 1201^**^
	SA300 UV 1	6,008 ± 653	53 ± 191^**^	3,647 ± 628
	USA300 PFA 1	6,235 ±1,716	53 ± 121^**^	3,309 ± 1,3701^**^
IL-23	USA300 live 0.1	1,008 ± 227	2 ± 11^*^	167 ± 411^*^
	USA300 HI 1	69 ± 39	26 ± 18	13 ± 7
	USA300 UV 1	481 ± 185	Not det	24 ± 24
	USA300 PFA 1	393 ± 321	20 ± 20	22 ± 15
IL-10	USA300 live 0.1	269 ± 139	3 ± 1.4	59 ± 24
	USA300 HI 1	6 ± 2	1.3 ± 1 3	3 ± 1.5
	USA300 UV 1	64 ± 29	1 ± 1	27 ± 15
	USA300 PFA 1	47 ±22	1 ± 0.7	17 ± 10
IL-8	USA300 live 0.1	18,575 ± 434	5,849 ± 1,6161^**^	14,994 ± 5,299
	USA300 HI 1	5,398 ± 1,270	6,162 ± 2,934	3,439 ± 749
	USA300 UV 1	6,366 ± 546	3,100 ± 972	3,826 ± 384
	USA300 PFA 1	5,308 ± 819	6,848 ± 3,666	4,057 ± 1,323
IL-1β	USA300 live 0.1	129 ± 41	4 ± 2	37 ± 8
	USA300 HI 1	4 ± 4	Not det	1 ± 1
	USA300 UV 1	14 ± 7	Not det	8 ± 4
	USA300 PFA 1	14 ± 6	2 ± 2	4 ± 2

DC were pre-treated with 5 μM Cytochalasin D or with 2 μM Chloroquine for 30 min and then stimulated for 24 h with live and differentially inactivated USA300 strain. The secretion of the indicated cytokines were measured in DC culture supernatants by Inflammatory Cytokine array kit. The results represent means ± SEM of three independent experiments (

* *p < 0.05*;

** *p < 001*;

*** *p < 0.001)*.

Thus, to determine if endosomal TLR signaling was involved in IFN-β induction, before stimulation with live or inactivated USA300, DC were treated with chloroquine, a weak base that prevents the endosomal acidification and impairs the signaling of TLRs localized in the endosome, as TLR3, TLR7/8, and TLR9. Chloroquine pre-treatment abolished IFN-β expression driven by live or HI *S. aureus* USA300, with little effect on IFN-β induced by UVI and PFAI bacteria. Similarly, phagocytosis and endosomal TLR-signaling inhibition also decreased IL-12, TNF-α, IL-6, and IL-10 production (Table 1). Conversely, the expression of the chemokine IL-8 was not inhibited by endosomal TLR signaling blockade (Table 1).

**Figure 6:**
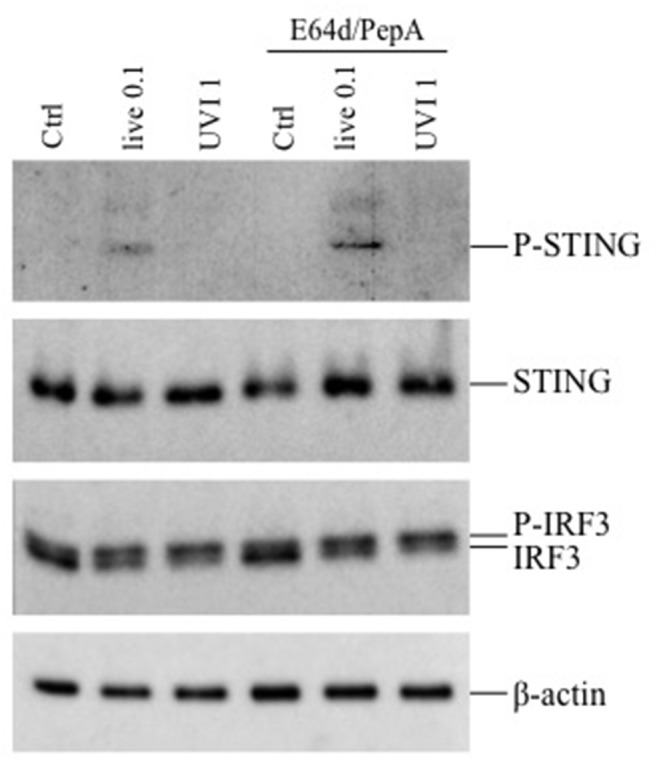
Activation of STING-IRF3 signaling pathway in response to live and UVI USA300. DC were left untreated (Ctrl) or stimulated with live and UVI USA300 strain for 5 h. Cell lysates were analyzed by Western blot to detect the expression and the phosphorylation of STING and IRF3. β-Actin levels were analyzed as control for protein loading.

Since the inhibition of phagosomal acidification by chloroquine impaired cytokine expression only in live or HI-stimulated DC, we postulated the involvement of other intracellular signaling pathways in UVI- and PFAI-stimulated DC. In particular, the activation of the cGAS–STING–IRF3 axis was previously shown to control IFN-β expression in murine and human macrophages infected with live *S. aureus* (13). Analysis of the STING/IRF3 pathway by Western blot revealed a faint band corresponding to phosphorylated STING only in live *S. aureus*-infected DC (Figure 6), whereas no STING phosphorylation was observed in UVI-treated cells despite IFN-β and IFN-α expression, which correlates with the appearance of a slower-migrating IRF3 phosphorylated form present in both live and UVI-stimulated DC (Figure 6). We also observed that inhibition of autophagy by E64D/PepA allowed the accumulation of phospho-STING in live *S. aureus*-infected DC (Figure 6), consistent with the observation that the autophagic molecule p62/SQSTM1 drives ubiquitinated STING to autophagosomes after its phosphorylation by TBK1 (31). Collectively, these data demonstrate that STING activation is likely required for IRF3 phosphorylation only in response to live *S. aureus* infection, thus suggesting the existence of a not fully characterized STING-independent and IRF3-dependent signaling pathway(s) in UVI-stimulated DC driving the IFN-β expression.

## Discussion

A full understanding of the complex interplay between *S. aureus* and the host immune responses represents a major goal for the development of an effective vaccine. Indeed, vaccine candidates tested to date have been ineffective in coping with the pathogenic complexity of *S. aureus* (32). Since vaccination relies on presentation of microbial antigens by antigen-presenting DC to naïve T cells, an understanding of the human DC response to *S. aureus* is essential for the development of preventive and therapeutic vaccine strategies.

In previous studies, we demonstrated that Esx virulence factors influence the DC response to *S. aureus* by modulating apoptosis, cytokine production, and, in turn, T cell differentiation (20). These data also demonstrated the utility of a DC-based *in vitro* model to study small differences in *S. aureus* virulence. Previously, it was shown that administration of heat-inactivated *S. aureus* failed to induce protection in mice, but rather skewed a beneficial IL-17 T cell response to a detrimental IL-10 producing T cell response (33). Also, immunization with *S. aureus* inactivated using UV irradiation, rather than HI, conferred protection to mice challenged with virulent methicillin-sensitive or methicillin-resistant strain by increasing survival and diminishing bacterial burden and kidney abscesses (34).

Based on these findings, we sought to investigate further the features of *S. aureus*–host interaction, focusing on the importance of both *S. aureus* viability and structural integrity in triggering DC immune responses. Our comparative analysis of the effects induced by live, HI, UVI, and PFAI USA300 sheds light on the capacity of DC to discriminate between live and inactivated *S. aureus*, and to fine-tune their responses according to the type and severity of the infectious threat (22). This feature mainly relies on the capacity of the immune cells to recognize the so-called *vita*-PAMPs, such as microbial RNA, bacterial metabolites, signaling molecules like second messengers, signal peptides (35), and quorum-sensing molecules (36), with the latter uniquely associated with live microorganisms (24).

Our analysis demonstrated that the different inactivation methods did not affect the capacity of USA300 to induce the expression of maturation markers, such as CD86, CD83, CD38, CD40, and HLA-DR; however, intriguing differences were found in the capacity of DC to drive the expansion of Th1 and Th17 producing CD4^+^ T cells in response to live or inactivated *S. aureus*. While both live and, at small extent, inactivated staphylococci induce IFN-γ production, only live USA300 promoted the expansion of IL-17 producing T cells, suggesting that Th1 and Th17 responses are induced by different mechanisms that might be independent (Th1) or dependent (Th17) from bacterial viability. However, the Th17 response seems to be related to the release of IL-1β, which occurs only from DC infected with live bacteria. IL-1β is a Th17-promoting cytokine that requires the proteolysis by caspase-1, for activation and secretion, which occurs only in response to live *S. aureus* infection (37).

These data prompted us to identify and further dissect similarity and divergence in cell response as well as intracellular pathways activated in human DC by either live *S. aureus* or inactivated bacterial preparations. For instance, the comparison of cytokine production induced by live, HI, UVI, and PFAI staphylococci revealed a different profile depending on the type of inactivation. Indeed, only UVI and PFAI *S. aureus* preserved the ability to induce in DC a robust production of IL-12, IL-6, and TNF-α. IL-23 was instead released only in response to live and UVI bacteria stimulation. Moreover, IL-1β, IL-10, and IL-8 were produced mainly by live USA300-infected DC. These data suggest that DC can discriminate between the risk associated with live bacterial infection *vs* the stimulation with an inactivated *S. aureus*. In particular, DC respond to stimulus with live bacteria producing, in addition to IL-12, IL-6, TNF-α, and IL-23, also IL-1β and IL-8, which are involved in neutrophil recruitment and early anti-staphylococcal defense in the invaded tissue (5). Moreover, to dampen an excessive activation of T cells and inflammatory phagocytes, which lead to host tissue damage, by recognizing live *S. aureus*, DC secrete the anti-inflammatory cytokine IL-10 that is able to influence disease outcome during acute infection (38). Surprisingly, IFN-β expression in DC stimulated with UVI and PFAI bacteria is higher than that found in DC infected with live *S. aureus*; conversely, the IFN-α expression displayed a time-dependent regulation where UVI bacteria resulted in a stronger inducer at an early time point such as 5 h compared to live *S. aureus* while the opposite occurs at later time point.

To further dissect the key events of host–pathogen interaction, we analyzed the contribution of *S. aureus* phagocytosis and internalization on cytokine expression. Phagocytosis was found to be an essential step for the activation of an optimal cytokine response to both live and inactivated USA300 bacteria. These data are consistent with previous experiments *in vivo* showing that *S. aureus* internalization was required for peritoneal macrophage cytokine response (39); furthermore, our observations indicate that the majority of inflammatory and regulatory signaling pathways occurred after bacterial internalization, based on the loss of cytokine expression in the presence of cytochalasin D. Although internalization is crucial for both live and inactivated USA300, it is likely that different intracellular pathways are triggered in response to either live or inactivated *S. aureus*. In particular, chloroquine treatment, which blocks endosomal acidification and subsequent endosomal TLR signaling, impacted the expression of IFN-β, as well as IL-12, TNF-α, and IL-6 in response to live and HI bacteria while it only poorly interferes with the UVI and PFAI *S. aureus*-induced expression of these cytokines. Interestingly, in mouse macrophages, both TLR and STING signaling pathways contributed to the transcriptional response induced by live but not killed *S. aureus* (13).

Having determined that IFN-β expression in UVI-stimulated DC was not dependent on endosomal TLR signal, we investigated the role of the STING/IRF3 axis; interestingly, STING activation was required for IRF3 phosphorylation only in response to live *S. aureus* infection, as previously observed (13). Conversely, IRF3 phosphorylation was induced in a STING-independent manner by UVI stimulation, suggesting the involvement of another unknown IRF3-dependent cytoplasmic sensor activated by UVI *S. aureus*.

Given the differences observed in cytokine production and Th response induced by the different bacterial inactivation procedures, an important aspect emerging from this study is the confirmation that high-temperature inactivation profoundly affects the immune stimulatory properties of *S. aureus*. Such observation could be related to profound alterations of PAMPs and structures in HI bacterium, which are essential to promote an appropriate DC response. Conversely, PFA and UV treatment preserved the *S. aureus* capacity to stimulate a range of cytokines that well overlapped with those released from DC infected with live bacteria with the exception of IL-10 and IL-1β. In addition, it is likely that the different killing methods may profoundly impact the ratio between *vita*- and classical PAMPs. Despite *S. aureus* replication, the availability and/or release of bacterial molecules involved in DC immune recognition may differ, depending on the inactivation method; for instance, UV-treated bacteria may preserve, for a while, some metabolic activities that trigger specific intracellular pathway(s) distinct from those induced by HI bacteria. Taken together, our results demonstrate that live or inactivated *S. aureus* infection stimulates distinct pathways in DC. Such information may prove helpful in the design and testing of novel vaccination strategies to optimize a protective immune response against this pathogen.

## Data Availability Statement

The raw data supporting the conclusions of this manuscript will be made available by the authors, without undue reservation, to any qualified researcher.

## Ethics Statement

The studies involving human participants were reviewed and approved by Istituto Superiore di Sanità - CE/13/387. The patients/participants provided their written informed consent to participate in this study.

## Author Contributions

MC performed experiments, analyzed data, and prepared the manuscript. SS performed experiments and analyzed data. ME performed experiments and participated in experimental design and data analysis. EG, RC, MS, and FR performed experiments. FB revised the manuscript and contributed to data interpretation. JH discussed the data. EC participated in experimental design, data analysis, and manuscript writing. All authors approved the manuscript before it was submitted.

### Conflict of Interest

FB is an employee of GSK Vaccines and owns patents on *S. aureus* vaccine candidates as well as GSK stocks. FB has no other relevant affiliations or financial involvement with any organization or entity with a financial interest in or financial conflict with the subject matter or materials discussed in the manuscript apart from those disclosed. The remaining authors declare that the research was conducted in the absence of any commercial or financial relationships that could be construed as a potential conflict of interest.
